# Establishing normal myocardial blood flow and myocardial flow reserve values: a rubidium-82 positron emission tomography study

**DOI:** 10.1007/s00259-026-07983-3

**Published:** 2026-06-06

**Authors:** Martin Lyngby Lassen, Niels Høeg Brandt-Jacobsen, Piotr Slomka, Tine W. Hansen, Yeliz Bulut, Caroline Kistorp, Andreas Kjaer, Philip Hasbak

**Affiliations:** 1 Department of Clinical Physiology and Nuclear Medicine, Rigshospitalet, Copenhagen, Denmark; 2 Cluster for Molecular Imaging, Department of Biomedical Sciences, Copenhagen University, Copenhagen, Denmark; 3 Division of Artificial Intelligence, Department of Medicine, Department of Cardiology, Cedars-Sinai Medical Center, Los Angeles, CA USA; 4 Steno Diabetes Center Copenhagen, Copenhagen, Denmark; 5 Department of Clinical Medicine, University of Copenhagen, Copenhagen, Denmark; 6 Department of Nephrology and Endocrinology, Copenhagen University Hospital- Rigshospitalet, Copenhagen, Denmark

**Keywords:** Rubidium-82, MBF, MFR, Normal material

## Abstract

**Purpose:**

Quantitative Rubidium-82 (^82^Rb) PET allows assessment of myocardial blood flow (MBF) and flow reserve (MFR), yet clinically applicable normal reference ranges remain incompletely defined across age and sex. This study aims to establish age- and sex-specific reference ranges for resting MBF (rMBF), stress MBF (sMBF), and MFR using ^82^Rb PET in healthy adults.

**Methods:**

Data from four cohorts of 277 carefully screened healthy adults (143 women, 134 men) aged 19–79 years were examined with an identical ^82^Rb PET protocol on the same scanner. Global rMBF, sMBF, and MFR were quantified with a 1-tissue compartment model. Age- and sex-specific percentiles were derived using natural spline regressions.

**Results:**

Median [IQR] rMBF was 1.00 [0.82; 1.18] mL/g/min, sMBF was 3.25 [2.82; 3.58] mL/g/min, and MFR was 3.18 [2.77; 3.86]. The rMBF increased with age (~ 1% per year), while sMBF and MFR decreased with age (sMBF ~-0.1%, MFR ~ -0.6% per year). Women had higher rMBF and sMBF, but lower MFR than men. Age- and sex-specific percentile curves (5–95%) demonstrated substantial physiologic variation in rMBF, sMBF, and MFR across the adult lifespan.

**Conclusion:**

This study establishes reference percentiles for rMBF, sMBF, and MFR derived from a uniform multicohort dataset of healthy adults, aged 19–79 years. These values may aid clinical interpretation of quantitative perfusion PET. These reference percentiles are intended to complement – rather than replace – validated prognostic thresholds for sMBF and MFR. As the population was almost exclusively Euro-Caucasian with body mass index in the normal to moderately overweight range, validation in more diverse cohorts is warranted.

**Supplementary Information:**

The online version contains supplementary material available at 10.1007/s00259-026-07983-3.

## Introduction

Rubidium-82 (^82^Rb) Positron Emission Tomography (PET) has emerged as a crucial tool for assessing regional ischemia caused by atherosclerosis. This modality focuses on quantifying resting and stress myocardial blood flow (rMBF and sMBF, respectively) and their ratio, the myocardial flow reserve (MFR), both of which sMBF and MFR serve as critical prognostic indicators [1–6]. An sMBF < 2.0 ml/g/min or MFR < 2.0 is indicative of adverse cardiovascular outcomes, thereby enhancing the stratification of cardiac risk in patients suspected of coronary artery disease (CAD) [4, 7]. While fixed thresholds for sMBF and MFR are well established for prognostic risk stratification, the purpose of age- and sex-specific reference percentiles is different. Reference percentiles provide physiological context for interpreting measured values in individual patients and may be particularly useful in settings such as suspected coronary microvascular dysfunction (e.g., INOCA/microvascular angina), diffuse atherosclerosis, and laboratory quality assurance/benchmarking. These percentiles should therefore not be interpreted as age- or sex-adjusted risk thresholds that would justify down-grading cardiovascular risk in older individuals with low flow values. The concurrent use of singular prognostic thresholds for both sMBF and MFR relies on data that adhere to well-defined, standardized parameters; however, recent research indicates that variables such as sex and age may significantly influence these metrics [8–12]. Consequently, establishing normal ranges of MBF and MFR while minimizing confounding variables is essential for refining current clinical assessments and potentially enabling personalized diagnostic and prognostic applications [11–14].

This study aimed to derive age- and sex-specific reference ranges for rMBF, sMBF, and MFR healthy adults using a single PET/CT system and standardized ^82^Rb infusion protocols.

## Materials and methods

### Study population

This study included healthy participants recruited from four cohorts, all of whom had no known indications of cardiovascular disease. Three of these cohorts have been extensively described in previous studies [15–17].

The first cohort included 26 healthy participants matched by age and sex to patients with type 2 diabetes. These individuals were enrolled between April and December 2013 and were required to be between 35 and 80 years of age, with no history or symptoms of cardiovascular disease, normal kidney function, and no treatment for pulmonary or cardiac conditions [15].

The second cohort consisted of 40 healthy young adults. These participants were enrolled from September 2016 to March 2017 as part of a randomized crossover study investigating the effect of caffeine on adenosine-induced myocardial vasodilation. Eligible participants were over 18 years of age, consumed coffee daily, had no diagnosed medical conditions, and had not used prescription medication, tobacco, or recreational substances within the preceding three months [16]. Of note, only the first of the repeated 0 mg caffeine (baseline) scans were included in this study.

The third cohort comprised 27 healthy, physically active men without a history of anabolic steroid use or cardiovascular disease. Recruitment took place between November 2021 and August 2023. Participants were required to be between 18 and 50 years of age, regularly engaged in recreational strength training, and have no current or past use of anabolic androgenic steroids or other performance-enhancing substances [17].

The fourth study cohort comprised 184 kidney donors, who were included retrospectively as a part of an institutional quality control at the Department of Nuclear Medicine, Rigshospitalet, Denmark (Capital Region of Denmark Research Legal Office (p-2024-18048)). The subjects underwent cardiac assessments with ^82^Rb-PET between January 2017 and December 2022.

To ensure a consistent definition of “healthy subjects”, all participants across the four cohorts were required to meet the same exclusion criteria. Exclusion criteria were comprehensive: (1) a history of CAD or other cardiovascular conditions, including stroke, angina, or dyspnea, confirmed through patient records and interviews; (2) a body mass index (BMI) exceeding 35 kg/m² ; (3) diabetes (defined as self-reported physician-diagnosed diabetes or use of glucose-lowering medications); (4) inability to understand study information; (5) bronchospastic lung disease, including Chronic Obstructive Pulmonary Disease (defined as self-reported physician-diagnosed COPD or use of COPD medications) or asthma (defined as self-reported physician-diagnosed asthma or use of asthma medications); and (6) current pregnancy or active breastfeeding.

All studies were conducted in accordance with the Declaration of Helsinki, complied with relevant Danish regulations, and received the relevant approvals. For the three prospective research cohorts, ethical approval was obtained from the Capital Region Committee on Health Research Ethics, and all participants provided oral and written informed consent prior to inclusion. For the retrospective kidney donor cohort, included as part of institutional quality assurance at the Department of Nuclear Medicine, Rigshospitalet, approval was granted by the Capital Region of Denmark Research Legal Office (p-2024-18048), which waived the requirement for informed consent.

### PET/CT imaging

Study participants underwent a rest/adenosine stress ^82^Rb myocardial perfusion imaging (MPI) protocol with a target dose of 30mCi (1110MBq). All MPIs were performed in a single 128-slice Siemens Biograph mCT PET/CT system (Siemens Healthineers, Knoxville, USA) using a 7-minute acquisition protocol. The acquisitions were obtained using the Bracco dose cart (Bracco Diagnostics), employing an infusion rate of 50mL/min. For the stress MPI, adenosine was infused continuously for 6 min (140 µg/kg/min), with the PET acquisition starting 2.5 min into the infusion [18]. The participants were instructed to abstain from substances and medications containing caffeine and theophylline for at least 12 h. The collected PET data were reconstructed into 18 dynamic frames (1 × 10 s, 8 × 5 s, 3 × 10 s, 2 × 20 s, 4 × 60 s), which were analyzed in QPET (Cedars-Sinai) using the Lortie Model [19]. Of note, the Lortie model utilizes a 1-tissue compartment model, providing K_1_ and k2 measures, with K_1_ representing the influx of ^82^Rb into the myocardial tissue. Given the non-linear and incomplete extraction of ^82^Rb during the first-pass of the radiotracer, the correlation between K_1_ and the true MBF is non-linear. This nonlinearity, also known as the “roll-off” effect, leads to an underestimation of the MBF at high flow rates, often observed with stress MPI [20]. All reconstructions were evaluated using interframe motion correction [21]. Coronary artery calcium score (CACS) was determined from a non-contrast CT scan acquired on the Siemens Biograph mCT using standard Agatston methodology as previously described [15]. Left ventricular ejection fraction (LVEF) at rest and during stress was derived from ECG-gated PET data using automated QGS software (Cedars-Sinai).

## Statistical analyses

We report the MBF, MFR, sex, and subject age for all individuals. rMBF, sMBF, and MFR did not adhere to a normal distribution; thus, the influence and modeling of the dependent variables (age and sex) were performed using natural splines to accommodate the non-linear relationships. The cohort provided continuous age coverage from 19 to 79 years; age was treated as a continuous variable throughout, and no stratum-specific subgroup analyses were performed. Furthermore, to test the stability of the model and to rule out potential influences of selection bias in the included cohorts, we performed spline analysis using both the whole cohort and a downsampled cohort excluding the respective cohorts, employing a leave-one-out method for the assessments. The results from the downsampled cohorts are shown in Supplementary Tables 1–3. The natural splines were chosen primarily due to their simplicity and stability in boundary analyses, and these types of splines tend not to overfit the data. Differences across sexes were performed using the Wilcoxon Rank-Sum test. Continuous variables are presented as medians and interquartile ranges (IQRs), given the non-normally distributed nature of the measured data. Categorical variables are presented as frequencies and corresponding percentages. Two-sided P-values < 0.05 were considered statistically significant. All analyses were performed using R (the GNU project).

## Results

This study comprised 277 individuals (143 (51%) were women) with a median (IQR) age of 53 (29–63) years and BMI of 24.3 (22.2–26.9) kg/m² (Table 1). Statistical assessments of the characteristics revealed comparable values across sexes.

**Table 1:** Characteristics. Numbers are given in % or median [1st ; 3rd quartiles] where appropriate

	Grouped, *N* = 277	Women, *N* = 142	Men, *N* = 135	Wilcoxon rank-sum test
Age (yr)	53 [29; 63]	55 [35; 64]	50 [27; 61]	0.247
BMI (kg/m^2^)	24.3 [22.2; 26.9]	24.0 [21.8; 26.9]	24.8 [22.9; 27.2]	0.151
Coronary Artery Calcium Score (Agatston score)	0 [0; 0]	0 [0; 0]	0 [0; 0]	0.561
Heart rate, Rest (bpm)	64 [58; 71]	65 [58; 72]	63 [56; 71]	0.367
Heart rate, Stress (bpm)	85 [75; 95]	85 [77; 96]	83 [73; 94]	0.021*
Systolic BP at rest (mmHg)	114 [104; 125]	113 [100; 125]	116 [105; 125]	0.311
Systolic BP at stress (mmHg)	112 [104; 122]	112 [103; 123]	113 [106; 122]	0.680
Rest RPP (bpm·mmHg)	7255 [6236; 8289]	7277 [6284; 8353]	7208 [5985; 8410]	0.942
Stress RPP (bpm·mmHg)	9702 [8175; 11007]	9711 [8353; 11158]	9477 [8008; 10922]	0.078
Rest LVEF (%)	66 [61; 71]	70 [66; 74]	63 [56; 71]	< 0.001*
Stress LVEF (%)	73 [67; 78]	77 [74; 80]	69 [65; 73]	< 0.001*
LVEF Reserve (%)	7 [4; 9]	7 [4; 9]	7 [5; 9]	0.544
Rest MBF (mL/g/min)	1.00 [0.82; 1.18]	1.10 [0.94; 1.28]	0.85 [0.74; 1.04]	< 0.001*
Stress MBF (mL/g/min)	3.25 [2.82; 3.58]	3.46 [3.14; 3.72]	2.95 [2.52; 3.30]	< 0.001*
MFR	3.18 [2.77, 3.86]	3.09 [2.68, 3.68]	3.36 [2.83, 4.06]	0.020*

### Resting MBF

**Fig. 1:**
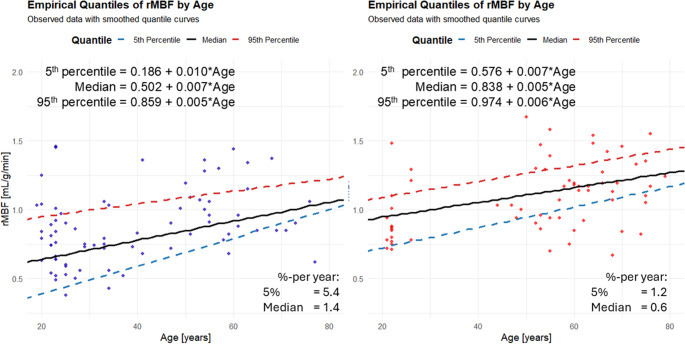
Resting MBF for men (left) and women (right) according to age

Significant sex – and age-dependencies were observed for the rMBF, with women exhibiting higher values than men (*p* < 0.001, Table 1). Spline-regressional analyses of the median and 5%/95% confidence intervals revealed a quasi-linear relationship between rMBF and age for both sexes (Fig. 1). The quasi-linear relationship estimated a median increase in rMBF of 1.4% per year for men and 0.6% per year for women, equaling a change in absolute measures of ~ 0.007 mL/g/min for men and ~ 0.005 mL/g/min for women (Table 2). Notably, the models remained stable when excluding data from the central part of the cohort (Cohort 4), whereas excluding cohorts that primarily comprised the young (Cohorts 2–3) and elderly (Cohort 1) populations revealed significant changes in the predicted normal rMBFs (Supplementary Table 1).

**Table 2:** Median and 5/95% percentiles for resting MBF [mL/g/min] for men and women per decade of life

Age (y)	5th % percentile	Median	95th percentile
Men			
20	0.39	0.64	0.95
30	0.49	0.71	1.00
40	0.59	0.78	1.04
50	0.69	0.85	1.09
60	0.79	0.92	1.14
70	0.90	0.98	1.18
80	1.00	1.05	1.22
Women			
20	0.72	0.95	1.09
30	0.80	1.00	1.15
40	0.87	1.05	1.20
50	0.92	1.09	1.26
60	1.00	1.14	1.30
70	1.09	1.21	1.38
80	1.14	1.25	1.42

### Stress MBF

**Fig. 2:**
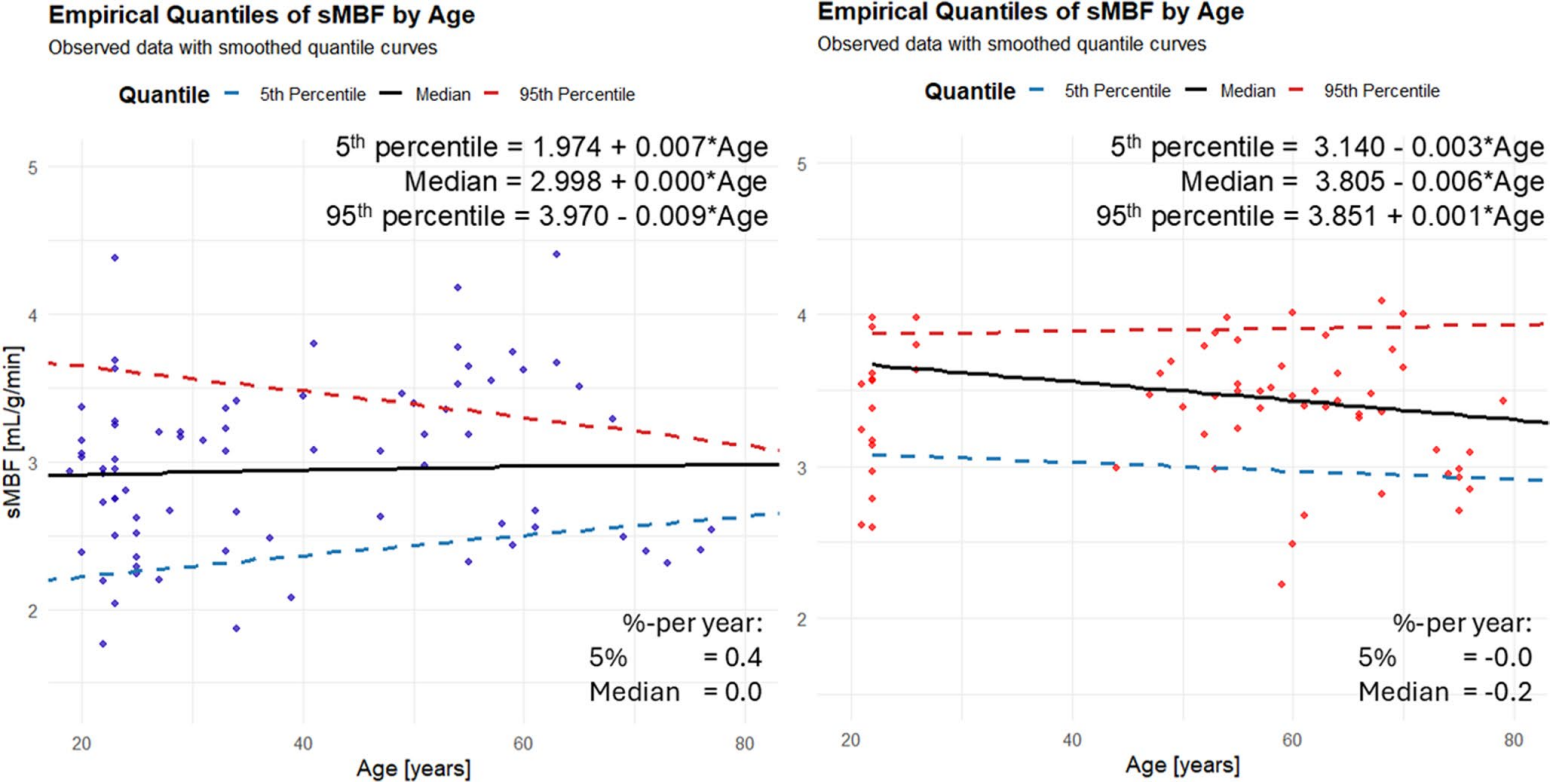
Stress MBF for men (left) and women (right) according to age

The sMBF was higher in women, compared to men (*p* < 0.001, Table 1). Spline-regressional analyses revealed that sMBF had a slight positive correlation to age for men, while women displayed a negative correlation (Fig. 2). Median increase in sMBF per year approximated ~ 0.001 mL/g/min for men and − 0.006 mL/g/min for women (Table 3). Similarly to the reports for the rMBF, we report the same influence of excluding the four cohorts using the leave-one-out method (Supplementary Table 2).

**Table 3:** Median and 5/95% percentile predicted intervals stress MBF [mL/g/min] for men and women, per decade of life

Age (y)	5th % percentile	Median	95th percentile
Men			
20	2.22	2.91	3.65
30	2.29	2.93	3.56
40	2.36	2.94	3.48
50	2.43	2.96	3.39
60	2.50	2.97	3.30
70	2.57	2.97	3.21
80	2.63	2.98	3.11
Women			
20	3.09	3.68	3.87
30	3.06	3.62	3.88
40	3.03	3.56	3.89
50	3.00	3.50	3.90
60	2.97	3.43	3.91
70	2.94	3.37	3.92
80	2.92	3.31	3.93

### MFR

**Fig. 3:**
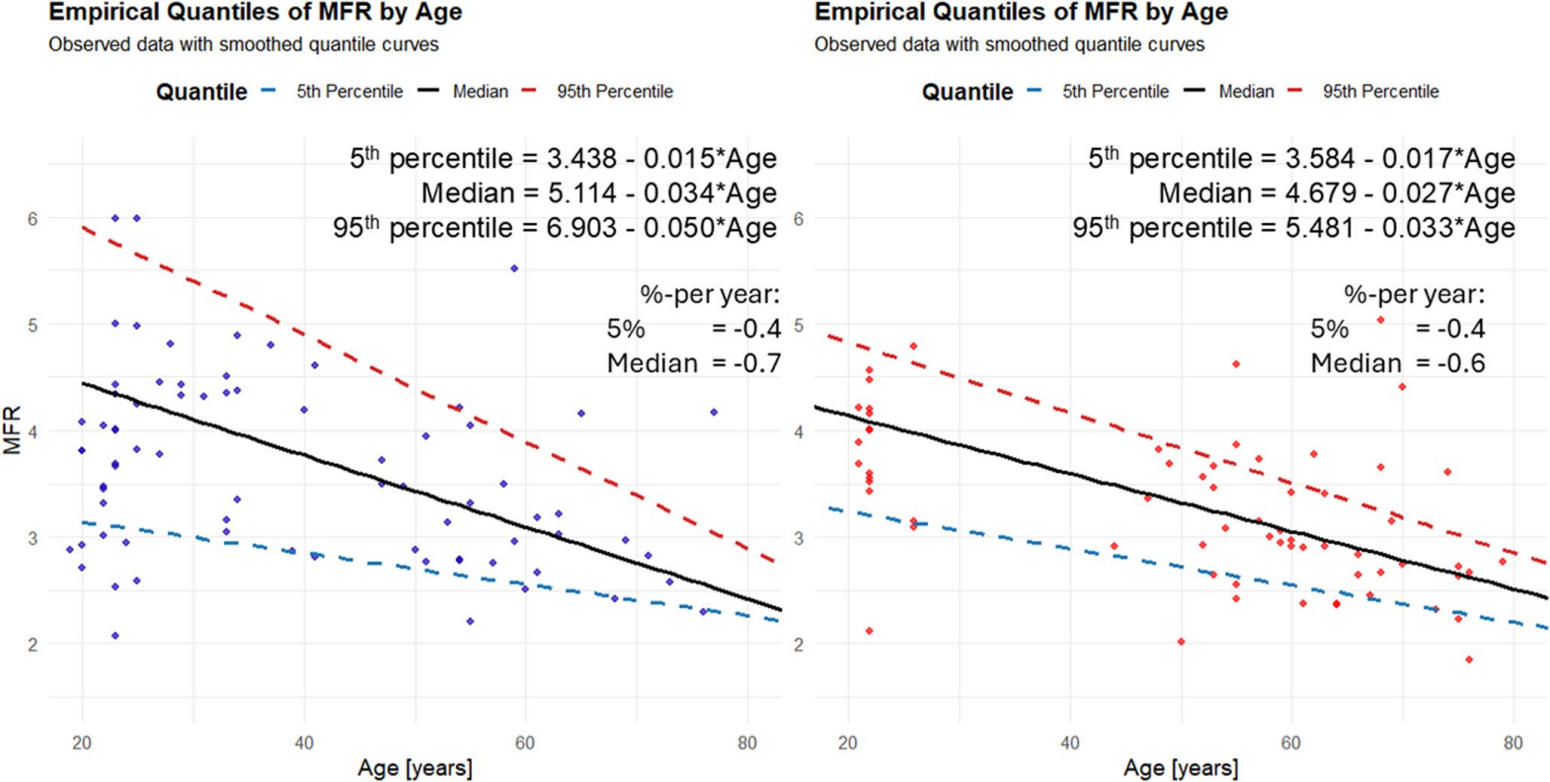
MFR for men (left) and women (right) according to age

Spline regression analyses revealed an inverse correlation between age and MFR for both women and men, corresponding to a relative decrease of ~ 0.6% per year (Fig. 3), corresponding to reductions in the MFR of ~ 0.03 per year in both men and women (Table 4). The predicted MFR using the leave-one-out method revealed that this technique is stable, regardless of the cohort left out of the model prediction.

**Table 4:** Median and 5/95% percentiles for predicted normal MFR obtained for men and women per decade of life

Age (y)	5th % percentile	Median	95th percentile
Men			
20	3.14	4.44	5.90
30	3.00	4.10	5.40
40	2.85	3.77	4.90
50	2.70	3.43	4.39
60	2.56	3.09	3.89
70	2.41	2.76	3.39
80	2.26	2.42	2.89
Women			
20	3.24	4.14	4.82
30	3.07	3.87	4.49
40	2.89	3.60	4.17
50	2.72	3.32	3.84
60	2.55	3.05	3.51
70	2.37	2.78	3.18
80	2.20	2.51	2.85

## Discussion

This study analyzed rMBF, sMBF, and MFR in 277 healthy individuals aged 19–79 years using ^82^Rb MPI scans. The primary findings highlight a dependency of age on the MBF/MFR, while also reporting sex-specific differences. The findings reported originate from a unique cohort of healthy participants with strict inclusion and exclusion criteria, which also included a uniform scanning protocol obtained on a single PET/CT system, a fixed ^82^Rb infusion rate, and consistent dosing. These strict criteria ensure a high reproducibility [8] and provide a robust reference for the normal hyperemic response to adenosine [22]. These age- and sex-specific percentiles provide a reference framework for interpreting quantitative perfusion PET. Percentile-based reporting may aid interpretation of borderline values, support evaluation of suspected coronary microvascular dysfunction/diffuse CAD by identifying unusually low values relative to a rigorously screened reference cohort, and facilitate laboratory quality assurance/benchmarking when acquisition and analysis pipelines are comparable. Importantly, values that are more common at older ages are not necessarily prognostically benign; validated thresholds for sMBF and MFR should continue to guide risk stratification. Consistent with this distinction, Danad et al. reported no improvement in diagnostic accuracy using age- or sex-specific cutoffs with ^15^O-H_2_O PET [23].

The modest positive association between stress MBF and age in men warrants specific comment, as it contrasts with findings from prior studies and with the age-trajectory observed in women. Several mechanistic explanations merit consideration. First, a survivor selection bias is plausible: in a rigorously screened healthy cohort, older men who remain asymptomatic and free of cardiovascular disease represent a highly selected subgroup. Men with subclinical microvascular dysfunction typically develop symptoms earlier and would have been excluded, leaving a cohort enriched for preserved vasodilatory capacity at older ages. This effect may be particularly pronounced in Cohort 4, where kidney donors undergo comprehensive pre-donation cardiovascular evaluation. Second, the divergent age-trajectories between sexes are mechanistically consistent with the accelerated loss of endothelium-dependent, oestrogen-mediated nitric oxide-dependent vasodilator function that occurs in women around and after the menopause transition [24]. The steep negative slope in women, by contrast, creates an apparent relative preservation or modest positive trend in men when analyzed in the same framework. Third, the absolute magnitude of the estimated male slope (~ 0.001 mL/g/min per year) is at the lower boundary of the ⁸²Rb PET test-retest repeatability [25, 26], and the leave-one-out sensitivity analyses indicate that stress MBF estimates in men are sensitive to cohort composition. Accordingly, this finding should be interpreted with caution and confirmed in future dedicated prospective studies with broader age coverage.

Of utmost importance, this study reports a positive correlation between age and rMBF, as well as negative correlations with sMBF and MFR; these findings are consistent with previously reported values by Sperry et al. [12] and a recent study evaluating the normal range of MBF and MFR in individuals with diabetes [27]. Moreover, our observations are supported by the DiaHeart study, which examined a large cohort of individuals with type 2 diabetes but without overt cardiovascular disease, using the same Siemens PET/CT system and identical ^82^Rb PET acquisition and quantification protocols [27]. In that study, women likewise exhibited higher resting and stress MBF but lower MFR compared with men, despite adjustment for traditional cardiovascular risk factors. This pattern is mathematically consistent with a ratio metric: although women demonstrated higher absolute hyperemic flow, they also had higher resting flow, such that MFR can be lower despite higher sMBF. Reporting both absolute flows and the ratio may therefore provide a more complete physiological picture. Taken together, the present data in healthy adults and the results from the DiaHeart study in individuals with diabetes suggest that sex-related differences in myocardial perfusion and flow reserve represent a physiological continuum, which may become clinically relevant when metabolic disease develops. Collectively, these studies emphasize the importance of accounting for both biological and methodological factors when interpreting MBF and MFR data across populations.

The observed association between age and rMBF should not be interpreted as an effect of age per se. Rather, it may be mediated by age-related changes in myocardial oxygen demand and ventricular-arterial coupling, including progressive central vascular stiffening and subtle concentric LV remodeling, which together increase wall stress and basal metabolic requirements [28–31]. In addition, age-related changes in sympathetic tone and respiratory work may contribute to higher resting demand. From a technical perspective, the observed increase in rMBF with advancing age may also be influenced by age-related changes in LV geometry. Specifically, increased concentricity and myocardial wall thickening can lead to a reduced partial volume effect. Since ^82^Rb-PET kinetic modeling relies on K_1_-based wash-in measurements, a reduction in the partial-volume effect would inherently result in higher measured flow values in thicker myocardium compared to younger subjects with thinner walls. Therefore, the age-dependent increase in rMBF might partially reflect these geometric alterations rather than being directly causative [32]. Because LV mass/concentricity measures were not uniformly available across cohorts, formal mediation analyses could not be performed.

In contrast, Sunderland et al. reported an opposite pattern, with a negative correlation between age and rMBF and a positive correlation between age and both sMBF and MFR, highlighting inter-study variability [10]. These discrepancies may stem from differences in the cohort characteristics, MBF/MFR quantification toolboxes, methodological approaches, and imaging protocols.

Importantly, the present study differs markedly from these previous investigations in several key aspects. Compared to Sperry et al., who studied a retrospective patient cohort with a substantial burden of cardiovascular risk factors (mean BMI 30.1 kg/m², 73% with angina-like symptoms, and high prevalence of hypertension and hyperlipidemia), our cohort consisted solely of rigorously screened healthy volunteers with no known cardiovascular disease or risk factors and a lower mean BMI of 24.3 kg/m². This distinction is critical, as lower MFR is well-documented in individuals with metabolic syndrome, increased BMI, hypertension, and dyslipidemia - all conditions that adversely affect coronary microvascular function [33–35]. In contrast to Sunderland et al., who recruited a smaller cohort of 49 healthy individuals aged 41–69 years, we included a broader age range (19–79 years) and a significantly larger sample size.

Differences in the methodological approaches for MBF assessment likely explain part of the inter-study variability. The use of different kinetic models is a key contributor, such as the one-tissue compartment modeling [19] and net retention [12] approaches, which yield systematically different absolute MBF and MFR values, limiting direct comparability across studies. Reported discrepancies between these methods may exceed 1 mL/min/g for stress MBF, corresponding to approximately 50% of the measured stress MBF, highlighting the importance of standardized quantification protocols when establishing reference ranges.

Additional variability arises from differences in vasodilator agents, PET/CT systems, and reconstruction protocols. Adenosine, regadenoson, and dipyridamole have distinct vasodilatory profiles, with dipyridamole generally producing lower hyperemic flow responses, which may contribute to differences in reported normal ranges [36–38]. Physiological variabilities, including heart rate, blood pressure, and other factors, which may affect accuracy, can be assessed using either short-term (within minutes) or intermediate-term (within days) test-retest studies. Such studies have reported variations in the MBF/MFR of up to 20% for ^82^Rb MPI [25, 26]. Moreover, the use of different PET/CT hardware introduces variation related to detector technology, time-of-flight performance, and correction algorithms; even within the same individuals, changes in PET systems can result in substantial differences in estimated kinetic parameters [39].

To assess internal robustness, we applied a “leave-one-out” approach, excluding individual cohorts. Model predictions for MFR remained stable across all analyses, while rMBF and sMBF were more sensitive to exclusion of cohorts representing the youngest and oldest age ranges (Supplementary Tables 1–3), highlighting the importance of broad age coverage when deriving normative values.

Because cohort composition influenced rMBF and sMBF estimates in sensitivity analyses, and because physical activity was not systematically measured across all cohorts, and exists on a continuous spectrum rather than discrete categories, we chose to present pooled reference ranges reflecting the diversity of healthy populations. This approach provides clinically applicable percentiles for general use, and the robustness of MFR across all sensitivity analyses, the most clinically relevant parameter for prognosis, supports the generalizability of our primary reference ranges.

This study has some additional limitations. The pooled reference population was derived from four cohorts recruited over a 10-year period, rather than from a single study designed to establish normal ranges. The 5th and 95th percentile estimates carry greater model uncertainty at the extremes of the age range (below age 30 and above age 70), where observational density is lower, and should be interpreted with particular caution. All participants underwent identical imaging on the same PET/CT system, and manufacturer-recommended quality control procedures were performed throughout the study period to minimize the risk of heterogeneous injection profiles and inter-scanner variation in image quality. The majority of participants were Euro-Caucasian with a BMI between 20 and 30 kg/m^2^, which may limit generalizability. Systematic documentation of traditional cardiovascular risk factors (including hypertension, dyslipidaemia, smoking, and obstructive sleep apnoea) was not uniformly recorded across all four cohorts. While blood pressure was measured during all examinations, CACS was zero in all participants, and cohort-specific eligibility criteria minimized the likelihood of significant undetected comorbidity, residual confounding from unmeasured risk factors cannot be entirely excluded. Future prospective studies establishing normative values should incorporate comprehensive risk factor phenotyping at the time of imaging. Menopausal status or hormonal therapy in women was not systematically recorded, which could contribute to observed sex differences. The use of a single PET/CT system and generators from one vendor may be both a strength and a limitation of this study. The strength is that variables in reconstruction protocols and differences in injection profiles are eliminated; however, the limitation is that the results may not be generally applicable to other systems or injection profiles. Finally, the stress MBF values at the 95%-percentile are near the upper limit of quantification for ^82^Rb; thus, significant roll-off effects are expected. Consequently, the stress MBF and, thus, the MFR may be underestimated in healthy subjects with adequate hyperemic responses to adenosine. However, this is a generalized problem across all ^82^Rb MPI assessments and thus a limitation of the imaging modality rather than of this study. These factors should be considered when applying the presented reference ranges in diverse patient populations.

## Conclusion

In a pooled cohort of rigorously screened healthy adults, we provide age- and sex-specific reference percentiles for rMBF, sMBF, and MFR using ^82^Rb MPI. The rMBF increased, while sMBF and MFR decreased, with advancing age; women demonstrated higher resting and stress MBF but lower MFR than men. These percentiles may support contextual interpretation of quantitative perfusion PET but are not intended to replace validated prognostic thresholds for sMBF and MFR. As the population studied was almost exclusively Euro-Caucasian with BMI in the normal to moderately overweight range, generalizability to other ethnic groups or extremes of body habitus should be confirmed in future studies.

## Supplementary Information

Below is the link to the electronic supplementary material.

Supplementary Material 1

## Data Availability

The datasets generated during and/or analyzed during the current study are available from the corresponding author on reasonable request.

## Abbreviations and acronyms

^82^Rb: Rubidium-82
PET: Positron Emission Tomography
CAD: Coronary Artery Disease
rMBF: rest Myocardial Blood Flow
sMBF: stress Myocardial Blood Flow
MFR: Myocardial Flow Reserve
MPI: Myocardial Perfusion Imaging
RPP: Rate Pressure Product
IQR: InterQuartile Range
HR: Heart Rate
SBP: Systolic Blood Pressure
BMI: Body Mass Index
