# Supplementary material for: Establishing normal myocardial blood flow and myocardial flow reserve values: a rubidium-82 positron emission tomography study
Source: Eur J Nucl Med Mol Imaging. 2026 Jun 6;53(10):6075–84. doi: 10.1007/s00259-026-07983-3 (PMC13421257; doi:10.1007/s00259-026-07983-3)
Supplement: Supplementary file 1 — Supplementary Material 1 [file 259_2026_7983_MOESM1_ESM.docx]

**Establishing normal myocardial blood flow and myocardial flow reserve values: A Rubidium-82 positron emission tomography study.**

Martin Lyngby Lassen, PhD^1,2^, Niels Høeg Brandt-Jacobsen MD^1^, PhD, Piotr Slomka, PhD^3^, Tine W Hansen MD, PhD ^4,5^, Yeliz Bulut, MD, PHD^6^, Caroline Kistorp MD, PhD^5,6^, Andreas Kjaer, MD, PhD, DMSc^1,2^, Philip Hasbak, MD, DMSc^1^

^1^Department of Clinical Physiology and Nuclear Medicine, Rigshospitalet, Copenhagen, Denmark.

^2^Cluster for Molecular Imaging, Department of Biomedical Sciences, Copenhagen University, Copenhagen, Denmark

^3^Division of Artificial Intelligence, Department of Medicine and Department of Cardiology, Cedars-Sinai Medical Center, Los Angeles, CA.

^4^ Steno Diabetes Center Copenhagen, Denmark

^5^ Department of Clinical Medicine, University of Copenhagen, Copenhagen, Denmark

^6^Department of Nephrology and Endocrinology, Copenhagen University Hospital- Rigshospitalet, Copenhagen, Denmark

Corresponding Author:
Martin Lyngby Lassen, PhD, Department of Clinical Physiology, Nuclear Medicine and PET and Cluster for Molecular Imaging, section 4011, Rigshospitalet and University of Copenhagen, Blegdamsvej 9, 2100 Copenhagen, Denmark

Tel: +45 35453520 Fax: +45 35454015

Email: [martin.lyngby.lassen@regionh.dk](mailto:martin.lyngby.lassen@regionh.dk)

**Supplementary Table 1.**

Median resting MBF [mL/g/min] using the leave-one-out method. Notably, the significant differences observed among younger adults when excluding [16] and [17] reflect the fact that these two cohorts comprise the majority of this age group, thereby significantly affecting the expected MBFs. Furthermore, the large deviations observed when excluding [15] for the 80-year-old subjects reflect that most of the subjects in this age group are from this cohort. Notably, the maximum percentage difference represents the absolute difference; the numbers in parentheses indicate the relative difference to the full cohort.

| Age (y) | All | Excluding [15] | Excluding [16] | Excluding [17] | Excluding Kidney Donors | Max % Diff to All |
| --- | --- | --- | --- | --- | --- | --- |
| Males | | | | | | |
| 20 | 0.64 | 0.62 (-3) | 0.74 (16) | 0.80 (25) | 0.68 (6) | 25 |
| 30 | 0.71 | 0.71 (0) | 0.78 (10) | 0.82 (15) | 0.74 (4) | 15 |
| 40 | 0.78 | 0.79 (1) | 0.83 (6) | 0.89 (14) | 0.79 (1) | 14 |
| 50 | 0.85 | 0.88 (4) | 0.88 (4) | 0.93 (9) | 0.85 (0) | 9 |
| 60 | 0.92 | 0.96 (4) | 0.93 (1) | 0.98 (7) | 0.90 (-2) | 7 |
| 70 | 0.98 | 1.05 (7) | 0.98 (0) | 1.02 (4) | 0.96 (-2) | 7 |
| 80 | 1.05 | 1.15 (10) | 1.03 (-2) | 1.07 (2) | 1.00 (-5) | 10 |
|  | | | | | | |
| 20 | 0.95 | 0.92 (-3) | 0.82 (-14) | N/A | 0.93 (-2) | 14 |
| 30 | 1.00 | 1.00 (0) | 0.90 (-10) | N/A | 0.99 (-1) | 10 |
| 40 | 1.05 | 1.06 (1) | 0.98 (-7) | N/A | 1.04 (-1) | 7 |
| 50 | 1.09 | 1.12 (3) | 1.06 (-3) | N/A | 1.09 (0) | 3 |
| 60 | 1.14 | 1.18 (4) | 1.14 (0) | N/A | 1.14 (0) | 4 |
| 70 | 1.21 | 1.24 (2) | 1.22 (1) | N/A | 1.20 (-1) | 2 |
| 80 | 1.25 | 1.30 (4) | 1.31 (5) | N/A | 1.25 (0) | 5 |

**Supplementary Table 2.**

Median stress MBF [mL/g/min] using the leave-one-out method. Notably, the significant differences observed for the younger adults when excluding [16] and [17] reflect the fact that these two cohorts comprise the majority of this age population, thus significantly affecting the expected MBFs. Furthermore, the large deviations observed when excluding [15] for the 80-year-old subjects reflect that most of the subjects in this age group are from this cohort. Notably, the maximum percentage difference represents the absolute difference; the numbers in parentheses indicate the relative difference to the full cohort.

| Age (y) | All | Excluding [15] | Excluding [16] | Excluding [17] | Excluding Kidney Donors | Max % Diff to All |
| --- | --- | --- | --- | --- | --- | --- |
| Males | | | | | | |
| 20 | 2.91 | 2.70 (-7) | 3.22 (11) | 3.11 (7) | 3.05 (5) | 11 |
| 30 | 2.93 | 2.88 (-2) | 3.21 (10) | 3.10 (6) | 2.98 (2) | 10 |
| 40 | 2.94 | 3.07 (4) | 3.15 (7) | 3.09 (5) | 2.90 (-1) | 7 |
| 50 | 2.96 | 3.34 (13) | 3.10 (5) | 3.07 (4) | 2.83 (-5) | 13 |
| 60 | 2.97 | 3.45 (16) | 3.05 (3) | 3.05 (3) | 2.75 (-8) | 16 |
| 70 | 2.97 | 3.63 (22) | 2.96 (0) | 3.02 (2) | 2.66 (-12) | 22 |
| 80 | 2.98 | 3.85 (29) | 2.87 (-4) | 2.97 (0) | 2.58 (-16) | 29 |
| Women | | | | | | |
| 20 | 3.68 | 3.58 (-3) | 3.79 (3) | N/A | 3.66 (-1) | 3 |
| 30 | 3.62 | 3.58 (-1) | 3.71 (2) | N/A | 3.61 (0) | 2 |
| 40 | 3.56 | 3.58 (1) | 3.62 (2) | N/A | 3.55 (0) | 2 |
| 50 | 3.50 | 3.58 (2) | 3.53 (1) | N/A | 3.50 (0) | 2 |
| 60 | 3.43 | 3.59 (5) | 3.46 (1) | N/A | 3.44 (0) | 5 |
| 70 | 3.37 | 3.59 (7) | 3.36 (0) | N/A | 3.38 (0) | 7 |
| 80 | 3.31 | 3.60 (9) | 3.27 (-1) | N/A | 3.32 (0) | 9 |

**Supplementary Table 3.**

Median MFR using the leave-one-out method. Notably, the maximum percentage difference represents the absolute difference; the numbers in parentheses indicate the relative difference to the full cohort.

| Age (y) | All | Excluding [15] | Excluding [16] | Excluding [17] | Excluding Kidney Donors | Max % Diff to All |
| --- | --- | --- | --- | --- | --- | --- |
| Males | | | | | | |
| 20 | 4.44 | 4.42 (0) | 4.24 (-5) | 4.20 (-5) | 4.36 (-2) | 5 |
| 30 | 4.10 | 4.11 (0) | 3.95 (-4) | 3.88 (-5) | 4.06 (-1) | 5 |
| 40 | 3.77 | 3.78 (0) | 3.66 (-3) | 3.59 (-5) | 3.75 (-1) | 5 |
| 50 | 3.43 | 3.30 (-4) | 3.34 (-3) | 3.30 (-4) | 3.45 (1) | 4 |
| 60 | 3.09 | 3.14 (2) | 3.07 (-1) | 3.01 (-3) | 3.14 (2) | 3 |
| 70 | 2.76 | 2.81 (2) | 2.77 (0) | 2.72 (-1) | 2.84 (3) | 3 |
| 80 | 2.42 | 2.49 (3) | 2.44 (1) | 2.40 (-1) | 2.54 (5) | 5 |
| Women | | | | | | |
| 20 | 4.14 | 4.18 (1) | 4.12 (0) | N/A | 4.21 (2) | 2 |
| 30 | 3.87 | 3.91 (1) | 3.85 (-1) | N/A | 3.93 (2) | 2 |
| 40 | 3.60 | 3.64 (1) | 3.58 (-1) | N/A | 3.65 (1) | 1 |
| 50 | 3.32 | 3.37 (2) | 3.32 (0) | N/A | 3.37 (1) | 2 |
| 60 | 3.05 | 3.10 (2) | 3.05 (0) | N/A | 3.09 (1) | 2 |
| 70 | 2.78 | 2.83 (2) | 2.79 (0) | N/A | 2.81 (1) | 2 |
| 80 | 2.51 | 2.56 (2) | 2.37 (-6) | N/A | 2.53 (1) | 6 |
